# Comparison of the pathogen species-specific immune response in udder derived cell types and their models

**DOI:** 10.1186/s13567-016-0307-3

**Published:** 2016-02-01

**Authors:** Juliane Günther, Mirja Koy, Anne Berthold, Hans-Joachim Schuberth, Hans-Martin Seyfert

**Affiliations:** Institute for Genome Biology, Leibniz Institute for Farm Animal Biology, Dummerstorf, Germany; Immunology Unit, University of Veterinary Medicine Hannover, Foundation, Bischofsholer Damm 15, 30173 Hannover, Germany

## Abstract

The outcome of an udder infection (mastitis) largely depends on the species of the invading pathogen. Gram-negative pathogens, such as *Escherichia coli* often elicit acute clinical mastitis while Gram-positive pathogens, such as *Staphylococcus aureus* tend to cause milder subclinical inflammations. It is unclear which type of the immune competent cells residing in the udder governs the pathogen species-specific physiology of mastitis and which established cell lines might provide suitable models. We therefore profiled the pathogen species-specific immune response of different cell types derived from udder and blood. Primary cultures of bovine mammary epithelial cells (pbMEC), mammary derived fibroblasts (pbMFC), and bovine monocyte-derived macrophages (boMdM) were challenged with heat-killed *E. coli*, *S. aureus* and *S. uberis* mastitis pathogens and their immune response was scaled against the response of established models for MEC (bovine MAC-T) and macrophages (murine RAW 264.7). Only *E. coli* provoked a full scale immune reaction in pbMEC, fibroblasts and MAC-T cells, as indicated by induced cytokine and chemokine expression and NF-κB activation. Weak reactions were induced by *S. aureus* and none by *S. uberis* challenges. In contrast, both models for macrophages (boMdM and RAW 264.7) reacted strongly against all the three pathogens accompanied by strong activation of NF-κB factors. Hence, the established cell models MAC-T and RAW 264.7 properly reflected key aspects of the pathogen species-specific immune response of the respective parental cell type. Our data imply that the pathogen species-specific physiology of mastitis likely relates to the respective response of MEC rather to that of professional immune cells.

**Electronic supplementary material:**

The online version of this article (doi:10.1186/s13567-016-0307-3) contains supplementary material, which is available to authorized users.

## Introduction

The outcome of a bacterial udder infection largely depends on the species of the invading pathogen. Gram negative bacteria, such as *Escherichia coli* elicit in most cases an acute severe inflammation with clinical signs which however may be self-healing by eventually eradicating the invader [1, 2]. Gram-positive bacteria, such as *Staphylococcus aureus* or *Streptococcus uberis* frequently cause only mild subclinical inflammations often allowing for persistent infections [3–6]. The molecular causes underpinning these quite substantial differences in pathogen species-specific mastitis are still unclear albeit those considerable experimental efforts that have been made during the last decade to decipher them. Several studies used transcriptome profiling of udder tissue retrieved from of cows having experimentally been infected with different pathogens. These studies revealed that *E. coli* infections elicit a strong cytokine storm [7, 8] while infections with *S. aureus* [9, 10] or *S. uberis* [11, 12] elicit a much weaker induction of proinflammatory cytokines.

Pathogens are perceived by pathogen recognition receptors (PRRs) from among which the toll-like-receptors (TLRs) form the best-characterized family. The ubiquitously expressed TLRs are activated through binding specific pathogen-derived molecular patterns (PAMPs) as ligands [13–15]. This event sets in train a signaling cascade ultimately leading to the activation of the NF-κB transcription factor complex. This serves as a master switch to regulate the expression of more than 200 different immune genes [16, 17].

Dissecting the molecular causes behind the pathogen species-specific immune physiology of mastitis requires appropriate model cells. In this regard it was established that the mammary epithelial cells (MEC) are highly relevant for both sentinel as well as effector functions of immune defense in the udder [18–20]. This cell type contributes to more than 70% of all cells from the lactating udder [21] and therefore might dominate the immune alert within-and emanating from-the udder early on after infection. Moreover, the pathogen species-specific activation profile of key immune genes in primary cultures of such cells (pbMEC) apparently reflects many aspects similar as recorded from in vivo infected udders [20, 22–26]. The SV-40 T transformed bovine MAC-T cell line [27] has frequently been used as an easy-to-handle MEC model for both, studying aspects of lactation and milk formation [27, 28] as well as for the analysis of immune functions of MEC [29–32].

Mammary epithelial cells line the alveoli in the milk parenchyma as a layer on top of myoepithelial cells, which are structurally supported by other cell types. These additional cells are initially also co-isolated during the procedure of purifying primary cultures of bovine MEC (pbMEC). In culture dishes they acquire an approximately spindle shaped cell morphology which is typical for fibroblasts. We will be referring to primary cultures hereof as primary bovine mammary derived fibroblast cultures (pbMFC). Skin derived fibroblasts from cows have recently been proven to featuring a considerable diagnostic potential for the immune competence of the cow [33, 34].

Professional immune cells, such as dendritic cells and macrophages also reside in the udder tissue [35] and these cells are known for their formidable capacity to synthesizing key cytokines [36]. Their quantitative contribution to calibrate the pathogen species-specific immune response in the udder early on after infection has not systematically been analyzed. Experimentally amenable models for macrophages may be established by differentiating bovine blood derived monocytes for several days in vitro (boMdM) [35]. Global transcriptome profiling of *S. aureus* infected boMdM suggested [37] that this infection triggered their alternative activation into a M2 phenotype associated with tissue remodeling rather than the M1 phenotype associated with acute inflammation (see [38] for a review on macrophage polarization).

Established murine macrophage model cell lines such as RAW 264.7 [39] or J774 [40] are more easily handled than boMdM. However, the fact that they are transformed through tumor viruses and that they stem from mouse rather than cattle sheds some doubts on the relevance of their use for modeling facets of immune regulation in the udder from cows. Interspecies comparisons of pathogen recognition may be of arguable value. Host species specific differentiated recognition of TLR4 ligands was proven by showing, for example that the lipid IVa variant of the LPS sub-fraction lipid A may act as TLR4 agonist in horse but as antagonist in human TLR4 signaling [41]. More examples have been documented [42] and X-ray crystallography revealed the structural basis for the host-species dependent PAMP recognition by TLR4 [42, 43]. Host-species dependent PAMP recognition was also shown for TLR2 and Dectin 1 [44].

We wanted to compare in pbMEC, primary fibroblast and macrophage model cells side-by-side the profile of the pathogen species-specific immune response, as elicited by challenges with *E. coli*, *S. aureus* and *S. uberis*. The direct comparison should validate and scale for the pbMEC the expected greatly different responses depending on the species of the challenging pathogen. Contrasting this profile with the response of the other cell types should allow to clearly identifying the very cell type governing the pathogen species-specific immune response in the udder early on after infection. Moreover, we wanted to scrutinize the usefulness of the easily handled MAC-T and RAW 264.7 cells to modeling key aspects of the MEC and macrophage specific and pathogen species-dependent immune functions.

We choose as a read out for immune functions the mRNA expression levels of a variety of key cytokine- and chemokine-encoding genes as parameters. These included TNF [45] and IL1A and IL1B [46] as well known key activators of inflammation and the pro- and anti-inflammatory IL6 as a master cytokine governing also the activation of the acute phase reaction [47–49]. We included a variety of chemokines since they are key players for the recruitment of immune cells [50]. CXCL2 and CXCL8 recruit PMNs to the site of infection [50, 51] while CCL5 attracts blood monocytes, memory T helper cells and eosinophils [52]. CCL20 was included, because this chemokine is not only attracting dendritic cells, as well as T- and B-cells [53] but has also some bactericidal properties against *E. coli* and *S. aureus* pathogens [54]. NOS2A [55] and the β-defensin LAP [56, 57] served as more classical biomarkers for bactericidal functions. Expression of *IL10* and the gene encoding the single-immunoglobulin interleukin-1 receptor-related (SIGIRR) served monitoring the modulation of anti-inflammatory pathways [58–60].

We found that the pbMEC reflects best key aspects of the pathogen species-specific mastitis and that both established model cell lines quite accurately mirror image key features of the pathogen species-specific characteristics of their respective parental cell type.

## Materials and methods

### Tissues, cells, cell line culturing and stimulation with mastitis pathogens

Tissues for the establishment of primary cultures of mammary epithelial cells (pbMEC) were retrieved from healthy first lactating Holstein Friesian heifers having been slaughtered at mid lactation in our local abattoir, complying with all pertinent ethical and legal requirements. The abattoir is an EU licensed (ES1635) core facility of the research affiliation and serves to routinely supply samples to different laboratories. Special ethical approval was unnecessary since the cows had been culled in the normal culling regime without conducting any animal experimentation.

Establishment of these cultures was essentially as described [61]. This reference describes also cultivation of the cells on collagen type I coated tissue plates (CELLCOAT, Greiner bio-one) in RPMI 1640 (Biochrom; Cat No F1215), having been supplemented with prolactin, dexamethasone, insulin and 10% FCS (PAN Biotech). The purification procedure of these cultures involves removal of fibroblasts by selective trypsinization. Such detached primary bovine fibroblast (pbMFC) cells were spun down (400 × *g*, 15 min) washed twice in PBS and subsequently cultivated on collagen coated tissue culture plates in the same medium as the pbMEC. MAC-T cells were cultivated in DMEM (Lonza) supplemented with 10% FCS on polystyrene tissue culture plates (CELLSTAR, Greiner bio-one). The mouse monocyte macrophage cell line RAW 264.7 (from ATCC) were cultivated in DMEM (Biochrom) supplemented with 2 mM l-glutamine and 10% FCS.

Establishment of the in vitro differentiated bovine monocyte-derived macrophages (boMDM) from the blood of lactating cows was previously described in detail [35]. Briefly, blood from healthy cows was drawn into heparinized vacutainer tubes from the *vena jugularis externa*. Mononuclear cells (MNC) were separated by density gradient centrifugation [35], suspended in MACS (magnetic-activated cell sorting) buffer (PBS, 2 mM EDTA) and labeled with paramagnetic MicroBeads™ coated with a CD14-specific monoclonal antibody (15 min, 4 °C; 20 µL beads and 80 µL MACS buffer per 1 × 10^7^ cells). MNC were washed in MACS buffer and subjected to MAC sorting. Positively selected CD14^+^ monocytes were suspended in RPMI 1640 culture medium (10% FCS) and labeled with PE-conjugated mouse anti-bovine CD14 antibody (1:50 in MACS buffer; ABD Serotec, Oxford, UK). Viability (≥98%) and purity (≥95%) of monocytes was flow cytometrically analyzed after addition of propidium iodide (2 µg/mL final). Cells were suspended in Iscové Medium (PAA, Pasching, Austria) supplemented with 10% (v/v) FCS and 1% (v/v) penicillin/streptomycin and cultured in 24 well plates (1 × 10^5^ cells/well) for 4 days at 37 °C and 5% CO_2_.

For challenge experiments, the cells were stimulated with 30 µg/mL of heat-killed *E. coli* strain 1303, *S. aureus* strain 1027, or *S. uberis* strain 233 particles for 1, 3, or 24 h. Unstimulated cultures served as controls. Heat-killed particles of *E. coli* strain 1303 and *S. aureus* strain 1027 were prepared as described [24]. *S. uberis* strain 233 [62] was grown in Todd Hewitt Broth (THB, Carl Roth GmbH) at 37 °C without agitation to the logarithmic phase of culture growth (0.5, OD_600_ nm). *S. uberis* pathogens were inactivated by heat treatment exactly as the *E. coli* or *S. aureus* mastitis pathogens (60 min, 80 °C). Based on three independent growth experiments, we found from exponentially multiplying cultures (OD_600nm_, 0.5) as protein content approximately 16.8 ± 4.1, 8.8 ± 1.2 and 5.7 ± 0.9 µg/10^7^ bacteria for of *E. coli*_1303_, *S. aureus*_1027_ and *S. uberis*_233_, respectively. Hence, application of 30 µg/mL of bacterial protein was approximately equivalent to MOIs of 10, 20 and 30 for *E. coli*, *S. aureus* and *S. uberis*, respectively.

### RNA extraction and mRNA quantification

RNA from pbMEC, MAC-T, pbMFC and RAW 264.7 was extracted with TRIZOL-reagent (Invitrogen). RNA from boMdM was extracted using the RNeasy Plus Micro Kit (Qiagen) according to instructions as provided in the manual. cDNA preparation (Superscript II, Invitrogen) and real time quantification of the mRNA concentrations with the Fast-Start Sybr Green I kit and the LightCycler II instrument (Roche) were done as detailed in [18], except that per assay 75 ng of total RNA was used as input. Relative copy numbers were titrated against external standards prepared from dilution series (10^6^–10 copies) of the cloned amplicons. They were also normalized across the different cell types against the amount the input of total RNA used for cDNA generation. Values from the MEC models pbMEC and MAC-T have in addition been separately normalized against copies of the not regulated CLIC1-encoding gene [63], with similar results as based on RNA input normalization. The RNA yield of from boMdMs was very limited. Hence, these data were normalized against copies from the GAPDH housekeeping reference gene. Sequences of oligo nucleotide primers are listed in Additional file 2.

### Determination of NF-κB activation

NF-κB activity was measured using a reporter gene expressing the Renilla-luciferase under the control of the NF-κB activated ELAM promoter (Invivogen; [61]). The reporter gene construct was transfected into pbMEC, MAC-T, and pbMFC with Lipofectamine 2000 (Invitrogen) as described [23]. RAW 264.7 cells are notorious for being difficult to transfect. Therefore we used the Neon^®^ Transfection System (Life Technologies) following the instructions of the manufacturer for this specific cell type. Briefly, 10^6^ cells were transfected with 5 µg reporter plasmid with one pulse of 1580 V for 20 ms. After transfection the cells were seeded in a 24-well plate and were allowed to recover overnight. Then they were challenged with the *E. coli*, *S. aureus* or *S. uberis* for 16 h, lysed and the luciferase activity was determined using the dual luciferase assay reporter system (Promega) as described [61]. The enzyme activity was calibrated against the protein content of the lysate rather than relative to the activity of a co-transfected thymidine kinase (TK) promoter driven luciferase expressing control plasmid. We have noted in earlier studies that activating the NF-κBp65 factor (as is the cases during induced TLR-signaling) may strongly quench the TK-promoter activity [64].

### Statistical analysis and data display

The data were analysed with GraphPad Prism Version 5 (GraphPad Software, Inc., La Jolla, CA, USA). Differences were evaluated through an analysis of variance (ANOVA) including Bonferroni’s correction for multiple pairwise comparisons. The criteria for statistical significance were fold change >2 and *P* < 0.05. Heat maps of gene expression were established with the Expander (EXPression ANalyzer and DisplayER) software [65].

## Results

Comparison of the immune competence and reactivity of different cell types has to address different levels. On the one hand, one needs to consider the basal gene expression levels in resting (unstimulated) cells. These contribute to shape the chemical environment in the surroundings. This might influence their neighboring cells or, in the case of MEC the concentration of bactericidal factors in the alveolar fluid, for example and thereby modulating the probability of manifestation of an infection. On the other hand, pathogen mediated modulation of gene expression represents a different key level of immune competence reflecting the capacity of the cell to respond to a given species of the attacking pathogen.

### Profile of basal expression level in RAW 264.7 differed grossly from the other model cells

**Figure 1:**
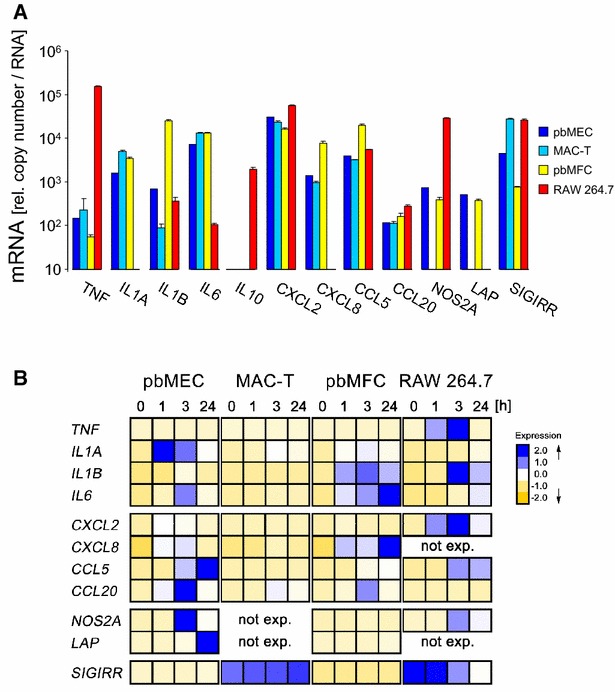
**Basal expression level of immune genes and its modulation after challenging with heat-killed** ***E. coli*** **.** **A** mRNA copy numbers relative to similar RNA inputs of TNF, IL6 and CCL20 as measured from the different cell types, as indicated. cDNA copy numbers were titrated against external standards and normalized according to the amount of RNA input. Note the broken ordinate in the graph of TNF. **B** Visualization of the data from several genes using the EXPANDER software. Each line displays the relative copy number of the respective gene as indicated over the time [h] of the challenge (0, 1, 3, 24), normalized across all cell types to the average of 0 and variance 1. Data are taken from Additional file 3. Data are mean values (error bars, ±SEM) from two replica experiments, each assayed in duplicate.

We profiled the expression levels of 12 immune genes in 4 of our 5 model cells (Figure 1A; Additional file 3 shows all data). For most genes they were quite similar between pbMEC, MAC-T and also pbMFC, with some exceptions. Key differences between pbMEC and MAC-T were that the latter cells did not express *NOS2A* and *LAP*, two of our parameters for bactericidal factors; and the level of the SIGIRR-encoding mRNA was almost tenfold enhanced in MAC-T compared to pbMEC. The primary cultures of fibroblasts (pbMFC) expressed both bactericidal genes similar as pbMEC, but a highly elevated (approximately 100-fold) basal concentration of IL1B-encoding mRNA distinguished their basal expression profile from pbMEC and MAC-T.

RAW 264.7 cells revealed a greatly deviating profile of basal gene expression. These cells uniquely expressed *IL10*, featured an almost 1000-fold increased concentration of the TNF-encoding mRNA and an approximately 40-fold higher concentration of the NOS2A-encoding mRNA than found in any of the other cells.

### Primary bovine MEC dominantly upregulated bactericidal effector genes after *E. coli* challenge

We challenged all our model cells with a strong stimulus of *E. coli* for recording the almost full extent of the cell type specific immune response. Therefore, primary cultures of bovine mammary epithelial cells (pbMEC) and mammary gland derived fibroblasts (pbMFC) were stimulated with 30 µg/mL of heat-killed particles from the mastitis causing *E. coli* strain 1303 for up to 24 h. The resulting modulation of the mRNA concentration of our candidate genes was measured. We compared these data with results from parallel challenge experiments using the established bovine MEC model cells MAC-T and the murine cell line RAW 264.7, as a widely used model for murine macrophages. The *E. coli* challenge increased in RAW 264.7 cells the already very high basal concentration of the TNF mRNA within 3 h by 80-fold (Figure 1B; Additional file 3) to eventually reaching >12 × 10^6^ copies per unit amount of RNA. The extent of increasing the TNF mRNA concentration was highest in pbMEC (>200-fold), but coming from a much lower basal level (148 ± 17 copies) of the control at t 0 h. It only reached approximately 3 × 10^4^ copies per unit amount of RNA as maximal concentration. Induction of the *TNF* levels was also significant in MAC-T and pbMFCs cells. However, the maximum levels reached by either of these cells were only 25 or 10% (MAC-T and pbMFC, respectively) of that as it was reached in pbMEC. RAW 264.7 cells synthesized also the highest mRNA concentrations of CXCL2 exceeding by fivefold the maximum concentration found in pbMEC.

The pbMFC turned out to be the dominant source for *IL6* and *CXCL8* messages (Figure 1; Additional file 3). The challenge increased the IL6 mRNA concentration in these cells initially with the same kinetic as in the epithelial cells. However, it was never downregulated in pbMFCs at later times during the challenge unlike as found in pbMEC. Rather, the IL6 mRNA concentration kept increasing in pbMFC with the duration of the challenge.

Distinguishing key features of the pbMEC were their ability to express highest levels of *IL1A*, *CCL5* and of the bactericidal genes after the *E. coli* challenge (Figure 1B). This was not only very clear for the well-known antimicrobial products from the β-defensin LAP and NOS2A-encoding genes but also for the bactericidal chemokine *CCL20*. Its expression increased by >1700-fold, 3 h after the *E. coli* stimulus (Additional file 3). These cells also revealed the highest induction (>1100-fold) for *NOS2A* expression, leading to a maximum mRNA concentration of more than 0.8 × 10^6^ copies per RNA equivalent. For comparison, RAW 264.7 reached less than 50% of that concentration and pbMFC only approximately 3% hereof.

### Only RAW 264.7 cells regulated the expression of the immune dampening factors IL10 and SIGIRR

Only RAW 264.7 cells significantly expressed *IL10* and the challenge increased this level by >tenfold during the first 3 h (Additional file 3). The increased expression of this dampening factor of inflammation was contrasted by the observed challenge mediated downregulation of the high basal levels of the SIGIRR mRNA concentration in the same cells (Figure 1B; Additional file 3). The basal level of the SIGIRR mRNA concentration in MAC-T cells was at similar high levels as found in RAW 264.7 cells but was not downregulated during the *E. coli* challenge.

### Gram-positive pathogens elicited a widespread immune alert only in professional immune cells

We compared the pathogen species-specific immune response of the different cell types by challenging them with heat-inactivated preparations of *S. aureus* strain 1027 and *S. uberis* strain 233 in parallel to the *E. coli* challenges. We added, as another cell model the response of monocyte-derived macrophages from cattle having been differentiated in vitro for 4 days (boMdM). This should allow to cross-checking the validity of conclusions drawn from the murine RAW 264.7 cells. We profiled the response of boMdM cultures established from three different cows (Additional files 1 and 5). Two of them responded quantitatively quite similar (#434, #561), while the cultures from the 3rd cow responded stronger and with faster induction of several genes. We included into the main comparison only the data from those similarly reacting cultures.

**Figure 2:**
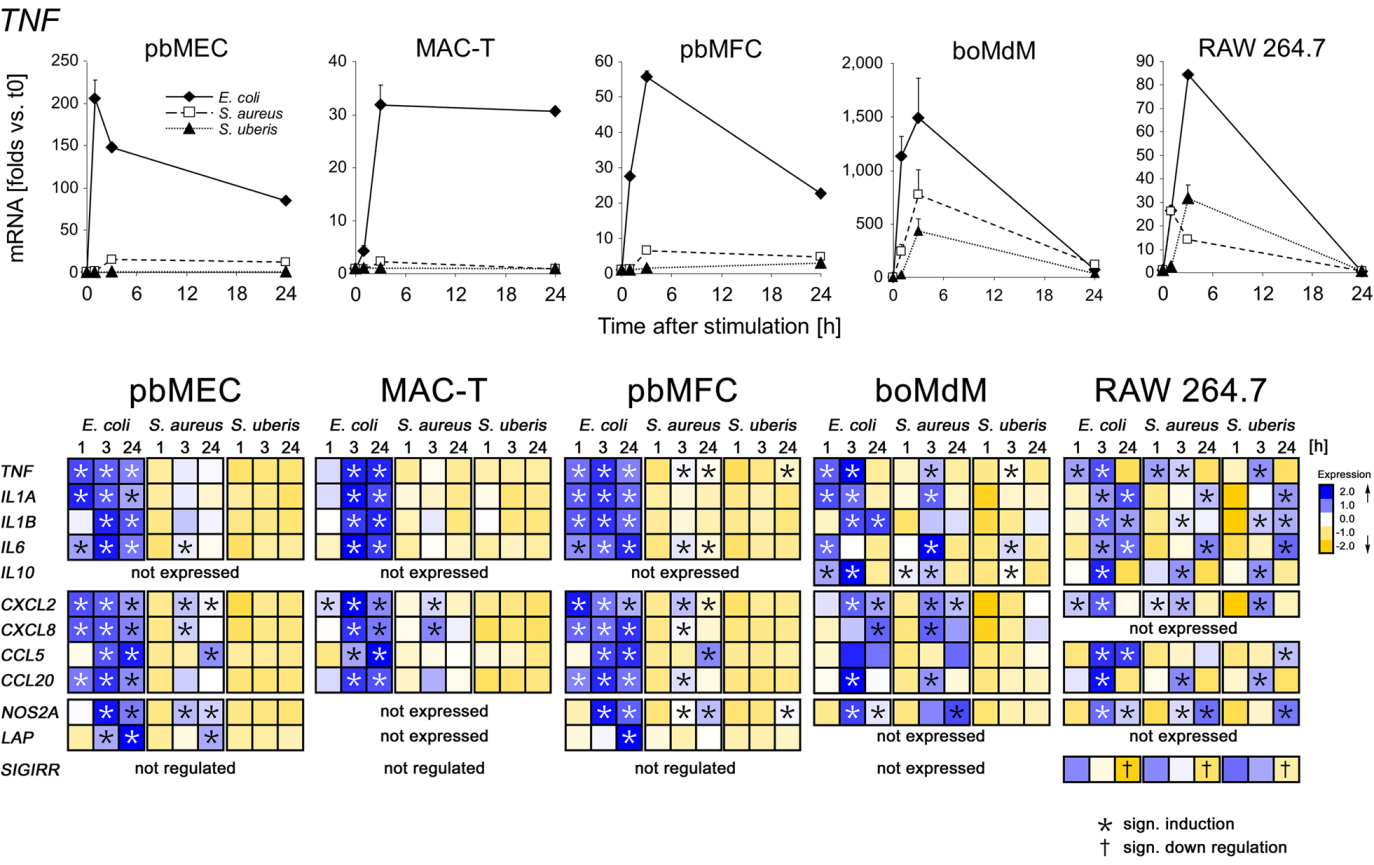
**Pathogen species-specific immune response of different cell types.** Upper panel: Changes in the level of *TNF* expression (ordinate) over time (abscissa) after challenging with heat-killed particles of the indicated pathogens. Lower panel: visualization of the data from several genes using the EXPANDER software. Each line displays the relative copy number of the respective gene as indicated over the time [h] of the challenge (1, 3, 24), normalized across all cell types to the average of 0 and variance 1. Data are taken from Additional file 4. Data are mean values (error bars, ±SEM) from two replica experiments, each assayed in duplicate.

The *E. coli* challenge maximally induced all the candidate genes, as expected (Figure 2; Additional file 4). The response against *S. aureus* was always stronger in the three cell types pbMEC, MAC-T and pbMFC than against *S. uberis*. Indeed, this pathogen did not induce any of the candidate genes to a significant extent in these cells. Maximum *S. uberis* caused gene inductions were recorded in pbMFC for *TNF* and *NOS2A* (3.1- and 4.5-fold; Additional file 4). All other *S. uberis* related gene inductions were well below twofold and statistically insignificant. In stark contrast, challenges with any of the three pathogens elicited in boMdM and RAW 264.7 a robust response characterized by a strong induction of immune gene expression. Again, induction of gene expression for most genes was strongest by *E. coli* and weakest by *S. uberis*, but the extent of inductions were all in the same order of magnitude for all genes (Figure 2).

### *S. aureus* and *S. uberis* activated NF-κB factors only in RAW 264.7 cells

**Figure 3:**
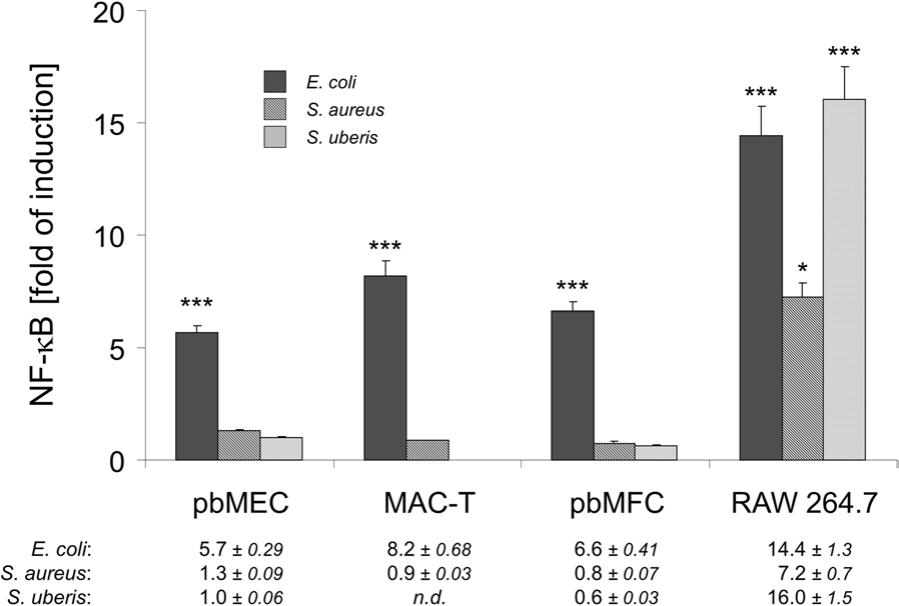
**Pathogen species-specific induction of NF-κB activity in different cells.** Cells were transiently transfected with the NF-κB reporter plasmid and stimulated with 30 µg/mL of protein from preparations of the heat-killed pathogens, as indicated. The increase in NF-κB activity was measured from cell lysates sampled 24 h after the challenge. Mean values from two independent experiments, each assayed in triplicate. *, *P* < 0.05; ***, *P* < 0.001.

Pathogen challenge induced activation of NF-κB factors serves as a master switch for the regulation of immune gene expression. It is also an integrating marker for any TLR-signaling. We monitored levels of active NF-κB by transfecting a NF-κB driven luciferase expressing reporter gene into those cells and subsequently challenging them with the respective pathogens. BoMdMs could not be included into these experiments due to their limited availability and their notorious poor transfection efficiency*. E. coli* strongly (4.5- to 14-fold) activated NF-κB factors in all 4 different cell types (Figure 3). In contrast, *S. aureus* and *S. uberis* activated NF-κB only in RAW 264.7 cells, but not in the models for epithelial cells (pbMEC, MAC-T) and supporting cells (pbMFC). Of note, *S. uberis* induced the level of active NF-κB factors in the RAW 264.7 cells at least as strongly as *E. coli*.

## Discussion

The udder is composed of a variety of different cell types each featuring a developmentally determined distinct immune competence. Their interplay governs the pathogen species-specific immune physiology of the udder early on after a bacterial infection. A central goal of our study was therefore to identify the very cell type of the udder whose pathogen species-specific immune response profile conforms best with the in vivo well documented divergent physiology of the pathogen species-specific of mastitis [4, 5]. This should validate the relevance of the respective cell type for modelling molecular aspects of mastitis physiology. Our second, more technical goal was to evaluate the relevance of the established cell lines MAC-T and RAW 264.7 for modeling mastitis relevant key immune functions in MEC and macrophages from cows. Using established cell lines has the advantage of reproducibly providing a homogenous cell population ensuring good technical repeatability of experiments. Primary cell isolates inherently reflect the individual variability between donors and variance eventually introduced during the purification and differentiation procedure. This is exemplified by our data regarding the quantitative (not qualitative) differences in the extent of immune stimulation of boMdMs through the challenges with the three pathogen species.

We have used heat-killed pathogens throughout. This allows monitoring under standardized conditions the passive—PAMP related—stimulation property triggering the initial immune response of the host cell. Our previous work has shown that challenging MEC with heat-killed *E. coli* very quickly (<1 h) activates NF-κB factors and cytokine gene expression [63]. This approach ignores the eventually crucial effects of virulence factors secreted by live pathogens. The influence of adherence and invasion upon the host cell response could also not be monitored in this experimental setting, since these properties are also intimately associated with functions of the live pathogens. However, using live pathogens in challenge experiments is technically demanding. Different pathogen species have quite different growth properties regarding both generation time as well as lag periods after re-inoculating cultures. Hence, experiments stimulating five different host cells with living cultures of three different pathogens are very difficult standardize. We have previously found no substantial difference in NF-κB and cytokine gene activation between short time (1 h) co-culture of MEC with live *E. coli* and *S. aureus* pathogens as compared to challenges using heat-killed preparations of the same pathogens [63]; the same was found comparing challenges with live vs. heat killed *S. uberis* [66]. This supports the value of using heat-killed pathogens in challenge experiments.

### Profiles of the cell type specific immune capacities

We have used a strong *E. coli* challenge [67] to revealing the full cell type specific immune response capacity of the various cell types. As distinguishing features of the MEC emerged their high capacity to expressing the bactericidal factors β-defensins and *CCL20* together with their pivotal capacity to express the cell recruiting factors *CXCL2*, *CXCL8* and *CCL5*. Their sustained capacity to express and secrete bactericidal factors obviously serves to directly fighting off bacteria and preventing colonization of the alveolus. The pathogen mediated induction of the PMN recruiting chemokines *CXCL2* and *CXCL8* was transient, while it was sustained for the monocyte recruiting factor *CCL5*. The only transient induction of PMN recruitment through MEC conceivably helps confining the danger of inducing immune pathology through overshooting secretion of aggressive factors from PMNs. This is particularly relevant considering the shear mass of MEC in the udder. In contrast, the cell types recruited by CCL5 are not known to secrete these very aggressive factors. The strong induction of *IL1A* gene expression in the MEC conceivably indicates that, upon injury related death of the MEC this factor is released into the surrounding as an inflammation mediator. It was shown that IL-1 may serve as a necrosis (but not apoptosis) related “damage-associated-molecular-pattern” capable of inducing sterile inflammation, for example during hypoxia [46].

The fibroblast pbMFC uniquely revealed after induction the sustained high level expression of *IL6* and *CXCL8*. Hence, these cells maintain secreting their danger induced signals and sustain their calling for help through cellular factors of innate immunity, since the invaded pathogens will not go away but rather keep multiplying at that specific location. However, they will contact only few cells in their immediate surrounding. This situation differs from that of epithelial cells lining the alveoli. Here, the pathogens are rapidly moving around conceivably contacting many cells and hence the risk of inducing an overshooting alarm must be avoided.

Most obvious features of the RAW 264.7 macrophage model cells was their extraordinary high capacity for expressing *TNF* and the neutrophil attracting factor *CXCL2*. Hence, activation and recruitment of macrophages to the site of infection multiplies by orders of magnitude the initial danger signals (*TNF*, *CXCL2*) emitted by the epithelial cells. The macrophage model cells were the only to modulate the expression of two, yet unrelated dampening factors of inflammation. Only RAW 264.7 and boMdM cells expressed *IL10* and stimulated its expression after pathogen stimulation. A prominent function of secreted IL10 is to confine the extent of inflammation by downregulating cytokine expression (among them IL1, IL6, TNF) in relevant target cells, such as TH1 cells [58, 68].

RAW 264.7 cells downregulated the expression of *SIGIRR* after pathogen stimulation. This factor is thought to interfere with TLR-signaling through preventing TLR-receptor dimerization. This prohibits formation of productive MyD88 dependent TLR-signaling [60]. Hence, downregulating the synthesis of this factor increases the sensitivity of the TLR-signaling cascade. *SIGIRR* expression serves also as a marker for differentiation since this factor is substantially expressed in monocytes, but only very weakly in fully differentiated macrophages [69].

### Similarities and differences between the parental cell types and their established models

Comparison of the pathogen species-specific profile of gene induction shows for all genes that MAC-T responded weaker than pbMEC, however with the same kinetic. Importantly, it reflected the same gradation of the response as pbMEC (*E. coli* > *S. aureus* > *S. uberis*) including the almost complete absence of an immune reaction against the *S. uberis* challenge. We have previously reported that the pbMEC response pattern against *S. aureus* strain 1027 is typical for several *S. aureus* strains [63] and show in a companion paper that their response against *S. uberis* strain 233 is typical for 20 different *S. uberis* strains, all having been isolated from udders of cows [66]. *E. coli* strain 1303 is representative for 21 other *E. coli* isolates from cases of both acute as well as persistent mastitis by the parameter of strong NF-κB activation in MAC-T cells (data not shown).

Moreover, we encountered in control experiments (unpublished) that different concentrations of FCS modulate the response of MAC-T cells similarly as reported from pbMEC [63]. Absence of NF-κB induction through an *S. aureus* challenge in pbMEC was identified as key determinant for their low level immune response against *S. aureus* [23, 24] and *S. uberis* [66]. This indicates that the challenge did not activate any TLR-mediated signaling. MAC-T cells reflect also this highly important key feature of the pathogen species-specific immune response of pbMEC. Hence, our data together validate that MAC-T cells reflect some of the most crucial features distinguishing the immune reaction of MEC from professional immune cells.

However, we note two key differences between both MEC models. First, MAC-T cells do not express the pivotal bactericidal β-defensin factors (*LAP* as an example) and *NOS2A*. We have previously observed that the capacity of MEC for expressing β-defensins is lost within 2 or 3 passages of pbMEC [19]. Hence, it represents a very sensitive marker for maintenance of the MEC phenotype and its loss in MAC-T cells indicates some degree of dedifferentiation. Second, the SIGIRR mRNA concentration was approximately tenfold higher in MAC-T than in pbMEC. This may attenuate TLR-signaling in MAC-T cells compared to pbMEC. *SIGIRR* expression was not modulated through pathogen stimulation, in neither of both MEC model cells.

The comparison of the reaction profile of boMdM and RAW 264.7 reveals that strong induction of the immune gene expression by all three pathogen species is the common and significant similarity between these two cell models. This is enabled by the strong activation of the NF-κB factor complex in these cells by all three pathogens. This suggests that they all triggered TLR-signaling in these cells. The approximately equal immune responsiveness against Gram-negative as well as Gram-positive pathogens appears to be an evolutionary conserved phenotype common to cells of the macrophage lineage. We concluded in our previous studies that MEC are obviously unable to unpack the relevant ligands of Gram-positive cells (hence lipoproteins) for activating productive TLR2 signaling, for example [63]. Macrophages, on the other hand are known as professional antigen presenting cells. They do have the capacity to internalize bacteria, kill them (as indicated by high basal *NOS2A* expression, for example) and processing them for immune recognition. Hence, diverse TLR-receptors and intra-cellular PRRs are likely to become activated by epitopes of Gram-positive bacteria which may not be recognizable by the trans-membrane TLR receptors [70].

However, we note three possibly significant differences between boMdM and RAW 264.7 cells. First, the extent of *TNF* induction was much stronger in boMdM than in RAW 264.7 cells. Second, *IL1A* and *IL6* expression was only transiently induced in boMdM while the increase in mRNA concentration was sustained in RAW 264.7 cells. Last, *SIGIRR* expression was absent in boMdM, while being high in RAW 264.7 cells. This validates that the boMdM had indeed been differentiated into macrophages [69].

Our study shows in summary that the models for mammary epithelial cells and fibroblasts, but not macrophages respond with distinctly graded immune reactions against each of the three pathogens. *E. coli* but neither of the Gram-positive bacteria elicits in them synthesis of a strong and transient cytokine storm. This distinction is in part caused by the failure of MEC to activate TLR-mediated signaling upon challenges with *S. aureus* or *S. uberis*. Hence, the pathogen species-specific norm of the immune response of MEC appears to dictate the immune response of the udder early on after infection. Our direct comparison also reveals that *S. uberis* elicits in MEC an even weaker induction of immune functions than *S. aureus*. Both established model cell lines, MAC-T and RAW 264.7 properly reflect most of these key features of pathogen species-specific immune response of the respective parental cell type.

## Additional files

10.1186/s13567-016-0307-3 **Pathogen specific regulated gene expression in primary bovine monocyte-derived macrophages (boMdM)**. Extent and kinetics of modulated mRNA expression of *TNF*, *IL6* and *CCL20* after stimulating boMdM from three different animals (#434, #561, #996) with *E. coli*_1303_, *S. aureus*_1027_ or *S. uberis*_233_ for various times. Values are means from two technical replicas (± SEM) of fold changes relative to unstimulated controls. Data are taken from Additional file 5.
10.1186/s13567-016-0307-3 **Sequences of the oligonucleotide primers used for real-time PCR quantification**. List of the primers used for RT-qPCR, of the pertinent source files and the resulting amplicon sizes.
10.1186/s13567-016-0307-3 **Modulated mRNA concentrations after stimulating pbMEC, pbMFC, RAW 264.7 or MAC-T with** ***E. coli***_**1303**_ **for various times to illustrate basic and full-scale mRNA expression in these cell types**. Values are means from two biological replica experiments, each assayed in duplicate (± SEM) of relative copy numbers; grey underlay, significant induction; red underlay, significant down regulation, fold change > 2, *P* < 0.05 vs. unstimulated control. Bonferroni’s correction for multiple analyses was applied.
10.1186/s13567-016-0307-3 **Extent and kinetics of modulated mRNA concentrations after stimulating pbMEC, pbMFC, RAW 264.7 or boMdM (#434 and #561) with** ***E. coli***_**1303**_, ***S. aureus***_**1027**_ **or** ***S.*** ***uberis***_**233**_ **for various times**. Values are means from two biological replica experiments, each assayed in duplicate (± SEM) of fold changes relative to unstimulated controls; grey underlay, significant induction; red underlay significant down regulation, fold change > 2, *P* < 0.05 vs. unstimulated control. Bonferroni’s correction for multiple analyses was applied.
10.1186/s13567-016-0307-3 **Pathogen specific regulated gene expression in primary bovine monocyte-derived macrophages (boMdM)**. Extent and kinetics of modulated mRNA concentrations after stimulating boMdM from three different animals (#434, #561, #996) with *E. coli*_1303_, *S. aureus*_1027_ or *S. uberis*_233_ for various times. Values are means from two technical replicas (± SEM) of fold changes relative to unstimulated controls; grey underlay, significant induction, fold change > 2, *P* < 0.05 vs. unstimulated control. Bonferroni’s correction for multiple analyses was applied.

## Abbreviations

CLIC1: chloride intracellular channel 1
GAPDH: glyceraldehyde 3-phosphate dehydrogenase
FCS: fetal calf serum
GAPDH: glyceraldehyde-3-phosphate dehydrogenase
MOI: multiplicity of infection
NF-κB: nuclear factor kappa-light-chain-enhancer of activated B-cells
PMN: polymorphnuclear granulocytes
PRR: pattern recognition receptor
TLR: toll-like receptor
RT-qPCR: reverse transcription quantitative PCR
