# Supplementary figures and images for: Comparison of the pathogen species-specific immune response in udder derived cell types and their models
Source: Vet Res. 2016 Feb 1;47:22. doi: 10.1186/s13567-016-0307-3 (PMC4736154; doi:10.1186/s13567-016-0307-3)

mRNA [folds vs. t0]

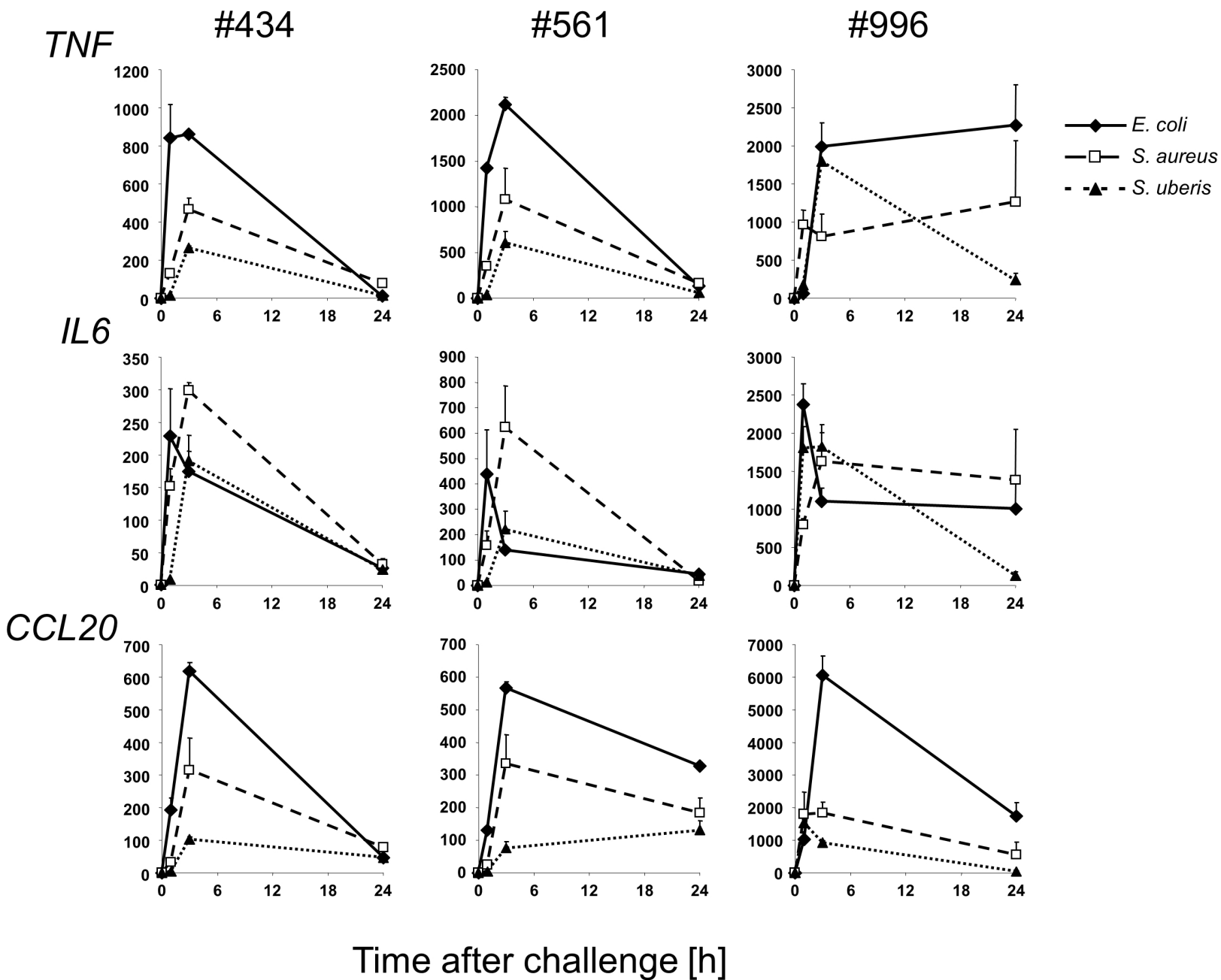

Supplement: Supplementary file 1 — 10.1186/s13567-016-0307-3 Pathogen specific regulated gene expression in primary bovine monocyte-derived macrophages (boMdM). Extent and kinetics of modulated mRNA expression of TNF, IL6 and CCL20 after stimulating boMdM from three different animals (#434, #561, #996) with E. coli 1303, S. aureus 1027 or S. uberis 233 for various times. Values are means from two technical replicas (± SEM) of fold changes relative to unstimulated controls. Data are taken from Additional file 5. [file 13567_2016_307_MOESM1_ESM.pdf]
