# Supplementary material for: Comparison of the pathogen species-specific immune response in udder derived cell types and their models
Source: Vet Res. 2016 Feb 1;47:22. doi: 10.1186/s13567-016-0307-3 (PMC4736154; doi:10.1186/s13567-016-0307-3)
Supplement: Supplementary file 2 — 10.1186/s13567-016-0307-3 Sequences of the oligonucleotide primers used for real-time PCR quantification. List of the primers used for RT-qPCR, of the pertinent source files and the resulting amplicon sizes. [file 13567_2016_307_MOESM2_ESM.docx]

**Additional file 2 Sequences of the oligonucleotide primers used for real-time PCR quantification.**

| **Species** | **Gene** | **GenBank no.** | **Primer sequence (5´–3´)** | **Amplificate size (bp)** |
| --- | --- | --- | --- | --- |
| ***Bos taurus*** | |  |  |  |
|  | *TNF* | NM_173966.2 | CTTCTGCCTGCTGCACTTCG * | 156 |
|  |  |  | GAGTTGATGTCGGCTACAACG * |  |
|  |  |  |  |  |
|  | *IL1A* | NM_174092 | GGCCAAAGTCCCTGACCTCT | 224 |
|  |  |  | CTGCCACCATCACCACATTC |  |
|  |  |  |  |  |
|  | *IL1B* | NM_174093.1 | AACCGAGAAGTGGTGTTCTGC | 167 |
|  |  |  | TTGGGGTAGACTTTGGGGTCT |  |
|  |  |  |  |  |
|  | *IL6* | NM_000600.3 | GGAGGAAAAGGACGGATGCT | 227 |
|  |  |  | GGTCAGTGTTTGTGGCTGGA |  |
|  |  |  |  |  |
|  | *IL10* | NM_174088.1 | GTGGAGAAGGTGAAGAGAGTC | 176 |
|  |  |  | CGTCATGGAGTCTAGTAGAGTC |  |
|  |  |  |  |  |
|  | *CXCL2* | NM_001046513.2 | GCCAAACCGAAGTCATAGCC | 213 |
|  |  |  | TGGAAACCAGCCATTCTCTTC |  |
|  |  |  |  |  |
|  | *CXCL8* | NM_173925 | CCTCTTGTTCAATATGACTTCCA | 170 |
|  |  |  | GGCCCACTCTCAATAACTCTC |  |
|  |  |  |  |  |
|  | *CCL5* | NM_175827 | TCCCCATATGCCTCGGAC | 229 |
|  |  |  | TCGCACCCACTTCTTCTCTG |  |
|  |  |  |  |  |
|  | *CCL20* | NM_174263.2 | CAGCAAGTCAGAAGCAAGCAA | 179 |
|  |  |  | CCCACTTCTTCTTTGGATCTGC |  |
|  |  |  |  |  |
|  | *NOS2A* | NM_001076799 | ACAGGATGACCCCAAACGTC | 188 |
|  |  |  | TCTGGTGAAGCGTGTCTTGG |  |
|  |  |  |  |  |
|  | *LAP* | NM_203435 | AGGCTCCATCACCTGCTCCTT | 182 |
|  |  |  | CCTGCAGCATTTTACTTGGGCT |  |
|  |  |  |  |  |
|  | *SIGIRR* | NM_001082443.2 | TGAGCAATGGAAGCCTCTATGA | 147 |
|  |  |  | AGCTGATGTTCCGGATGGAG |  |
|  |  |  |  |  |
|  | *CLIC1* | NM_001015608 | GTCTCAGTCCGCCTCTTGGT | 153 |
|  |  |  | AGAACAACCGCAGGTCGAAT |  |
|  |  |  |  |  |
|  | *GAPDH* | NM_001034034.2  XM_001252511.5 | CATTGACCTTCACTACATGGT | 232 |
|  |  |  | ACCCTTCAAGTGAGCCCCAG |  |
| ***Mus musculus*** | |  |  |  |
|  | *TNF* | NM_013693.3  NM_001278601.1 | ccgtcagccgatttgctatc | 160 |
|  |  |  | agttggtcccccttctccag |  |
|  |  |  |  |  |
|  | *IL1A* | NM_010554.4 | GACTACAGTTCTGCCATTGAC | 155 |
|  |  |  | CGGCTCTCCTTGAAGGTGAA |  |
|  |  |  |  |  |
|  | *IL1B* | NM_008361.4 | ACGGACCCCAAAAGATGAAG | 112 |
|  |  |  | GCCTGCCTGAAGCTCTTGTT |  |
|  |  |  |  |  |
|  | *IL6* | NM_031168.1 | AACGATGATGCACTTGCAGA | 195 |
|  |  |  | CTGAAGGACTCTGGCTTTGTC |  |
|  |  |  |  |  |
|  | *IL10* | NM_010548.2 | CCTGGGTGAGAAGCTGAAGAC | 140 |
|  |  |  | TGGCCTTGTAGACACCTTGG |  |
|  |  |  |  |  |
|  | *CXCL2* | NM_009140.2 | actctcaagggcggtcaaaa | 191 |
|  |  |  | caggtacgatccaggcttcc |  |
|  |  |  |  |  |
|  | *CCL5* | NM_013653.3 | CCCTCACCATCATCCTCACT | 181 |
|  |  |  | CGAGTGACAAACACGACTGC |  |
|  |  |  |  |  |
|  | *CCL20* | NM_001159738.1  NM_016960.2 | GCAGCAAGCAACTACGACTG | 180 |
|  |  |  | AGCCCTTTTCACCCAGTTCT |  |
|  |  |  |  |  |
|  | *NOS2A* | NM_010927 | CTTGCCCCTGGAAGTTTCTCT | 183 |
|  |  |  | GAACATTCTGTGCTGTCCCAGT |  |
|  |  |  |  |  |
|  | *SIGIRR* | NM_023059.3 | CGTGAAGAGGTCCCAGAAGAG | 135 |
|  |  |  | AGAACACCCAAGCTGTGCAA |  |
|  |  |  |  |  |
|  |  |  |  |  |
|  | *upper line: forward-, lower line, reverse-primer | | |  |
