# Supplementary material for: Comparison of the pathogen species-specific immune response in udder derived cell types and their models
Source: Vet Res. 2016 Feb 1;47:22. doi: 10.1186/s13567-016-0307-3 (PMC4736154; doi:10.1186/s13567-016-0307-3)
Supplement: Supplementary file 3 [file 13567_2016_307_MOESM3_ESM.docx]

Additional file 2 Modulated mRNA concentrations after stimulating pbMEC, pbMFC, RAW 264.7 or MAC-T with *E. coli*$_{1303}$ for various times to illustrate basic and full-scale mRNA expression in these cell types. Values are means from two biological replica experiments, each assayed in duplicate (± SEM) of relative copy numbers; grey underlay, significant induction; red underlay significant down regulation, fold change > 2, $P < 0.05$ vs. unstimulated control. Bonferroni's correction for multiple analyses was applied.

Gene	Time [h]	pbMEC	MAC-T	pbMFC	RAW 264.7
TNF	0	148 ± 17	229 ± 181	56 ± 6	151,534 ± 8,811
	1	30,417 ± 3,158	972 ± 238	1,534 ± 60	3,991,508 ± 322,009
	3	21,920 ± 357	7,311 ± 843	3,118 ± 81	12,788,609 ± 248,117
	24	12,642 ± 82	7,023 ± 47	1,270 ± 58	95,305 ± 2,773
IL1A	0	1,572 ± 61	5,059 ± 274	3,461 ± 239	10 ± 0
	1	146,035 ± 7,685	9,599 ± 490	28,403 ± 654	439 ± 42
	3	77,012 ± 1,521	27,039 ± 799	38,188 ± 126	6,363 ± 305
	24	29,845 ± 3,521	20,140 ± 1,614	25,559 ± 421	22,214 ± 2,273
IL1B	0	680 ± 54	88 ± 22	25,118 ± 1,600	367 ± 83
	1	5,387 ± 359	352 ± 67	175,108 ± 3,722	24,360 ± 1,984
	3	133,109 ± 41,428	3,449 ± 471	230,097 ± 699	327,595 ± 7,798
	24	59,581 ± 9,332	4,415 ± 348	158,874 ± 2,412	154,425 ± 17,778
IL6	0	7,183 ± 82	13,114 ± 303	12,999 ± 678	104 ± 8
	1	61,584 ± 3,010	13,666 ± 240	166,384 ± 4,340	377 ± 6
	3	277,552 ± 905	40,404 ± 58	246,868 ± 5,635	66,517 ± 5,492
	24	99,654 ± 7,785	32,805 ± 3,663	499,296 ± 8,141	160,099 ± 7,943
IL10	0	not expressed	not expressed	not expressed	1,965 ± 166
	1				2,184 ± 96
	3				22,597 ± 536
	24				388 ± 7
CXCL2	0	30,079 ± 515	23,121 ± 1,914	16,249 ± 895	55,778 ± 2,212
	1	1,616,908 ± 5,573	57,999 ± 924	724,305 ± 9,986	3,738,569 ± 308,329
	3	1,347,207 ± 27,859	175,526 ± 16,396	267,466 ± 2,766	8,807,634 ± 320,197
	24	626,861 ± 17,282	84,362 ± 3,233	142,889 ± 4,431	1,908,417 ± 126,017
CXCL8	0	1,399 ± 70	973 ± 53	7,813 ± 575	not expressed
	1	129,746 ± 10,380	5,785 ± 812	166,156 ± 4,794	
	3	144,236 ± 1,388	41,558 ± 4,364	150,372 ± 964	
	24	68,877 ± 6,825	21,366 ± 78	309,484 ± 6,946	
CCL5	0	3,883 ± 185	3,230 ± 26	19,424 ± 1,709	5,426 ± 36
	1	19,663 ± 837	2,979 ± 68	41,567 ± 2,021	3,901 ± 77
	3	733,381 ± 13,383	9,104 ± 1,190	340,700 ± 2,073	956,161 ± 50,464
	24	2,098,405 ± 133,856	29,816 ± 334	432,567 ± 23,660	828,457 ± 2,735
CCL20	0	114 ± 9	112 ± 10	160 ± 30	273 ± 27
	1	39,969 ± 3,993	2,206 ± 214	15,280 ± 444	543 ± 4
	3	202,896 ± 3,280	34,577 ± 5,795	84,198 ± 272	3,810 ± 295
	24	27,021 ± 3,389	21,474 ± 1,088	20,541 ± 1,326	144 ± 5
NOS2A	0	730 ± 23	not expressed	386 ± 51	28,835 ± 763
	1	10,325 ± 867		698 ± 11	31,483 ± 2,080
	3	835,279 ± 5,409		21,955 ± 569	327,086 ± 29,139
	24	114,504 ± 4,446		9,613 ± 93	148,310 ± 4,415
LAP	0	503 ± 42	not expressed	379 ± 20	not expressed
	1	539 ± 47		324 ± 3	
	3	4,470 ± 42		444 ± 29	
	24	59,483 ± 2,059		3,346 ± 32	
SIGIRR	0	4,539 ± 82	27,790 ± 581	761 ± 15	25,824 ± 1,837
	1	4,346 ± 229	29,800 ± 2,958	742 ± 29	25,363 ± 157
	3	4,311 ± 28	30,661 ± 732	842 ± 61	15,428 ± 907
