# Supplementary material for: Comparison of the pathogen species-specific immune response in udder derived cell types and their models
Source: Vet Res. 2016 Feb 1;47:22. doi: 10.1186/s13567-016-0307-3 (PMC4736154; doi:10.1186/s13567-016-0307-3)
Supplement: Supplementary file 4 [file 13567_2016_307_MOESM4_ESM.docx]

Additional file 4 Extent and kinetics of modulated mRNA concentrations after stimulating pbMEC, pbMFC, RAW 264.7 or boMdM (#434 and #561) with *E. coli*$_{1303}$, *S. aureus*$_{1027}$ or *S. uberis*$_{233}$ for various times. Values are means (from two biological replica experiments, each assayed in duplicate) ± SEM of fold change relative to unstimulated control; grey underlay, significant induction; red underlay significant down regulation, fold change > 2, $P < 0.05$ vs. unstimulated control. Bonferroni's correction for multiple analyses was applied.

		pbMEC			MAC-T			pbMFC			boMdM			RAW 264.7		
**Gene**	**Time**	***E. coli***	***S. aureus***	***S. uberis***	***E. coli***	***S. aureus***	***S. uberis***	***E. coli***	***S. aureus***	***S. uberis***	***E. coli***	***S. aureus***	***S. uberis***	***E. coli***	***S. aureus***	***S. uberis***
TNF	1	205 ± 21.3	2.2 ± 0.3	1.0 ± 0.0	4.2 ± 1.0	0.9 ± 0.0	1.1 ± 0.5	27 ± 1.1	1.3 ± 0.1	1.0 ± 0.0	1,132 ± 183.0	241 ± 64.0	24 ± 6.4	26 ± 2.1	26 ± 0.3	2.6 ± 0.1
	3	148 ± 2.4	15 ± 1.0	1.3 ± 0.1	32 ± 3.7	2.2 ± 0.3	1.0 ± 0.3	56 ± 1.5	6.6 ± 0.1	1.6 ± 0.1	1,492 ± 364.3	774 ± 227.3	435 ± 111.3	84 ± 1.6	14 ± 0.5	32 ± 5.5
	24	85 ± 0.6	13 ± 0.4	1.2 ± 0.1	31 ± 0.2	0.9 ± 0.2	1.0 ± 0.2	23 ± 1.0	4.8 ± 0.1	3.1 ± 0.3	73 ± 36.0	120 ± 29.8	36 ± 13.1	0.6 ± 0.0	0.7 ± 0.0	0.7 ± 0.0
IL1A	1	93 ± 4.9	2.0 ± 0.1	1.9 ± 0.2	1.9 ± 0.1	1.3 ± 0.1	1.4 ± 0.1	8 ± 0.2	1.3 ± 0.1	1.1 ± 0.0	119 ± 18.7	60 ± 6.4	7.9 ± 1.3	42 ± 4.0	17 ± 0.9	1.7 ± 0.4
	3	49 ± 1.0	8.1 ± 0.2	1.5 ± 0.0	5.3 ± 0.2	1.2 ± 0.2	0.8 ± 0.0	11 ± 0.0	1.9 ± 0.0	1.4 ± 0.0	110 ± 9.9	113 ± 21.3	55 ± 8.4	608 ± 29.1	82 ± 10.1	114 ± 31.2
	24	19 ± 2.2	3.4 ± 0.1	1.0 ± 0.0	4.0 ± 0.3	0.9 ± 0.1	0.9 ± 0.1	7 ± 0.1	1.5 ± 0.1	1.0 ± 0.0	48 ± 12.0	53 ± 14.9	54 ± 14.7	2,122 ± 217.1	339 ± 22.0	617 ± 65.2
IL1B	1	7.9 ± 0.5	0.9 ± 0.0	0.9 ± 0.0	4.0 ± 0.8	1.9 ± 0.3	4.3 ± 2.4	7.0 ± 0.1	1.2 ± 0.1	1.1 ± 0.0	165 ± 18.6	50 ± 2.9	6.1 ± 1.1	66 ± 5.4	28 ± 0.2	2.5 ± 0.2
	3	196 ± 61.0	15 ± 0.5	1.0 ± 0.1	39 ± 5.4	5.5 ± 0.8	1.0 ± 0.1	9.2 ± 0.0	1.8 ± 0.0	1.4 ± 0.0	474 ± 73.3	317 ± 43.4	105 ± 15.7	892 ± 21.2	175 ± 19.6	254 ± 57.7
	24	88 ± 13.7	7.4 ± 0.4	0.7 ± 0.1	50 ± 4.0	1.0 ± 0.2	1.0 ± 0.3	6.3 ± 0.1	1.4 ± 0.1	1.0 ± 0.0	482 ± 171.1	284 ± 79.4	268 ± 89.5	420 ± 48.4	142 ± 0.0	347 ± 35.3
IL6	1	8.6 ± 0.4	0.7 ± 0.1	0.6 ± 0.1	1.0 ± 0.0	0.9 ± 0.0	0.9 ± 0.0	13 ± 0.3	0.9 ± 0.1	0.7 ± 0.0	335 ± 97.8	155 ± 26.0	11 ± 2.5	3.6 ± 0.1	1.4 ± 0.0	0.9 ± 0.2
	3	39 ± 0.1	4.0 ± 0.3	1.2 ± 0.1	3.1 ± 0.0	1.2 ± 0.2	1.0 ± 0.0	19 ± 0.4	4.9 ± 0.2	1.3 ± 0.0	157 ± 16.9	461 ± 115.1	206 ± 34.6	638 ± 52.7	23 ± 1.4	10 ± 3.0
	24	14 ± 1.1	2.6 ± 0.0	1.1 ± 0.0	2.5 ± 0.3	1.0 ± 0.1	0.9 ± 0.0	38 ± 0.6	2.4 ± 0.0	1.1 ± 0.0	36 ± 7.8	26 ± 7.3	33 ± 5.6	1,537 ± 76.2	870 ± 54.7	1,603 ± 190.1
IL10	1										12 ± 1.0	9.2 ± 0.9	3.0 ± 0.5	1.1 ± 0.0	1.7 ± 0.2	0.9 ± 0.1
	3		not expressed			not expressed			not expressed		20 ± 2.3	11 ± 1.6	7.8 ± 1.9	11.5 ± 0.3	3.5 ± 0.4	3.7 ± 0.6
	24										4.3 ± 1.1	4.4 ± 0.7	3.3 ± 0.6	0.2 ± 0.0	0.2 ± 0.1	0.4 ± 0.0
CXCL2	1	54 ± 0.2	1.4 ± 0.0	0.6 ± 0.0	2.5 ± 0.0	1.7 ± 0.2	1.2 ± 0.0	45 ± 0.6	1.1 ± 0.0	0.7 ± 0.0	156 ± 12.7	84 ± 11.3	8 ± 0.7	67 ± 5.5	55 ± 0.5	4.2 ± 0.2
	3	45 ± 0.9	13 ± 0.3	1.5 ± 0.0	7.6 ± 0.7	2.5 ± 0.4	1.1 ± 0.0	16 ± 0.2	7.6 ± 0.4	1.2 ± 0.1	223 ± 16.0	205 ± 25.1	71 ± 9.1	158 ± 5.7	78 ± 4.4	109 ± 22.1
	24	21 ± 0.6	6.9 ± 0.1	1.2 ± 0.0	3.6 ± 0.1	1.5 ± 0.1	1.0 ± 0.0	9 ± 0.3	2.1 ± 0.0	1.1 ± 0.0	188 ± 35.3	176 ± 21.8	139 ± 22.1	34 ± 2.3	23 ± 1.3	25 ± 1.6
CXCL8	1	93 ± 7.4	1.1 ± 0.1	0.6 ± 0.1	5.9 ± 0.8	5.0 ± 1.2	1.3 ± 0.3	21 ± 0.6	0.9 ± 0.0	0.8 ± 0.0	26 ± 3.2	18 ± 2.1	6.1 ± 1.0			
	3	103 ± 1.0	15 ± 0.4	1.1 ± 0.1	43 ± 4.5	19 ± 2.7	1.7 ± 0.2	19 ± 0.1	4.1 ± 0.1	0.9 ± 0.0	42 ± 3.4	54 ± 8.9	22 ± 2.1		not expressed	
	24	49 ± 4.9	6.9 ± 0.0	1.0 ± 0.1	22 ± 0.1	7.3 ± 0.3	1.4 ± 0.0	40 ± 0.9	2.1 ± 0.0	1.0 ± 0.1	54 ± 21.4	47 ± 7.9	39 ± 9.6			
CCL5	1	5.1 ± 0.2	1.0 ± 0.0	1.2 ± 0.2	0.9 ± 0.0	1.3 ± 0.1	1.4 ± 0.0	2 ± 0.1	1.0 ± 0.1	1.0 ± 0.0	65 ± 10.2	8.1 ± 0.7	7.5 ± 4.1	0.7 ± 0.0	0.8 ± 0.0	0.7 ± 0.0
	3	189 ± 3.4	1.4 ± 0.0	1.1 ± 0.2	2.8 ± 0.4	1.5 ± 0.1	1.3 ± 0.1	18 ± 0.1	1.2 ± 0.0	1.0 ± 0.0	256 ± 35.1	45 ± 8.6	23 ± 11.5	176 ± 9.3	2.4 ± 0.1	1.7 ± 0.3
	24	540 ± 34.5	62 ± 12.8	0.9 ± 0.0	9.2 ± 0.1	1.7 ± 0.2	0.9 ± 0.0	22 ± 1.2	9.1 ± 0.1	1.2 ± 0.0	199 ± 69.9	207 ± 33.6	80 ± 11.6	153 ± 0.5	10 ± 2.2	17 ± 0.2
CCL20	1	352 ± 35.1	1.5 ± 0.3	1.0 ± 0.1	20 ± 1.9	3.9 ± 0.4	0.7 ± 0.1	95 ± 2.8	1 ± 0.0	0.9 ± 0.0	161 ± 23.5	28 ± 4.4	3.8 ± 0.7	2 ± 0.0	1.3 ± 0.0	1.0 ± 0.1
	3	1,784 ± 28.8	59 ± 5.0	1.0 ± 0.0	309 ± 52	39 ± 5.0	0.9 ± 0.1	526 ± 1.7	18 ± 1.1	0.8 ± 0.0	593 ± 19.9	326 ± 53.7	89 ± 12.1	14 ± 1.1	4.6 ± 0.3	3.7 ± 1.2
	24	238 ± 29.8	19 ± 2.7	1.2 ± 0.1	192 ± 9.7	7.1 ± 0.0	1.2 ± 0.1	128 ± 8.3	5 ± 0.3	1.0 ± 0.1	187 ± 81.4	131 ± 35.7	89 ± 27.2	1 ± 0.0	0.4 ± 0.0	0.4 ± 0.0
NOS2A	1	14 ± 1.2	1.3 ± 0.0	1.4 ± 0.0				1.8 ± 0.0	1.0 ± 0.1	0.9 ± 0.1	10 ± 2.2	6.5 ± 0.8	2.6 ± 0.2	1.1 ± 0.1	0.8 ± 0.1	0.8 ± 0.1
	3	1,144 ± 7.4	49.6 ± 12.9	1.2 ± 0.1		not expressed		56.9 ± 1.5	4.0 ± 0.4	1.0 ± 0.1	80 ± 18.2	64 ± 8.8	18 ± 3.0	11.2 ± 1.0	4.6 ± 0.4	2.2 ± 0.2
	24	157 ± 6.1	31.8 ± 3.3	1.0 ± 0.0				24.9 ± 0.2	8.4 ± 0.4	4.5 ± 0.5	42 ± 14.7	78 ± 19.6	19 ± 11.5	5.1 ± 0.2	10.9 ± 1.2	10.0 ± 0.2
LAP	1	1.1 ± 0.1	0.9 ± 0.1	1.0 ± 0.1				0.9 ± 0.0	0.9 ± 0.1	0.7 ± 0.0						
	3	8.9 ± 0.1	2.1 ± 0.1	1.0 ± 0.2		not expressed		1.2 ± 0.1	0.7 ± 0.0	0.7 ± 0.1		not expressed			not expressed	
	24	118 ± 4.1	9.0 ± 0.1	1.3 ± 0.1				8.8 ± 0.1	1.1 ± 0.1	0.8 ± 0.0						
SIGIRR	1	1.0 ± 0.1	1.0 ± 0.0	1.0 ± 0.0	1.1 ± 0.1	1.2 ± 0.1	1.3 ± 0.1	1.0 ± 0.0	1.1 ± 0.0	1.0 ± 0.0				1.0 ± 0.0	1.0 ± 0.1	1.1 ± 0.0
	3	0.9 ± 0.0	1.3 ± 0.1	1.0 ± 0.0	1.1 ± 0.0	1.1 ± 0.1	1.2 ± 0.0	1.1 ± 0.0	1.4 ± 0.0	1.1 ± 0.0		not expressed		0.6 ± 0.0	0.7 ± 0.0	0.9 ± 0.0
