# Supplementary material for: Comparison of the pathogen species-specific immune response in udder derived cell types and their models
Source: Vet Res. 2016 Feb 1;47:22. doi: 10.1186/s13567-016-0307-3 (PMC4736154; doi:10.1186/s13567-016-0307-3)
Supplement: Supplementary file 5 [file 13567_2016_307_MOESM5_ESM.docx]

Additional file 5 Pathogen specific regulated gene expression in primary bovine monocyte-derived macrophages (boMdM). Extent and kinetics of modulated mRNA concentrations after stimulating pbMdM from three different animals (#434, #561, #996) with *E. coli*$_{1303}$, *S. aureus*$_{1027}$ or *S. uberis*$_{233}$ for various times. Values are means from two technical replicas (± SEM) of fold changes relative to unstimulated controls.

			E. coli			*S. aureus*			*S. uberis*	
**Gene**	**Time**	**#434**	**#561**	**#996**	**#434**	**#561**	**#996**	**#434**	**#561**	**#996**
TNF	**1**	**841** ± *175.5*	**1,424** ± *9.7*	**57** ± *3.6*	**131** ± *4.8*	**350** ± *22.7*	**962** ± *199.4*	**14** ± *1.6*	**35** ± *6.2*	**175** ± *16.2*
	**3**	**864** ± *17.0*	**2,121** ± *78.4*	**1,988** ± *318.3*	**468** ± *58.5*	**1,079** ± *346.0*	**806** ± *297.4*	**265** ± *8.9*	**606** ± *127.2*	**1,801** ± *1.0*
	**24**	**11** ± *0.4*	**135** ± *11.4*	**2,269** ± *533.6*	**78** ± *3.1*	**163** ± *41.3*	**1,265** ± *806.7*	**14** ± *4.5*	**59** ± *4.0*	**236** ± *85.1*
IL1A	**1**	**91** ± *22.1*	**146** ± *6.6*	**221** ± *27.8*	**50** ± *6.4*	**70** ± *0.9*	**451** ± *133.7*	**5.8** ± *0.0*	**10** ± *1.2*	**390** ± *17.2*
	**3**	**97** ± *8.5*	**124** ± *12.1*	**345** ± *62.7*	**85** ± *15.0*	**142** ± *29.4*	**210** ± *36.0*	**48** ± *2.1*	**61** ± *18.0*	**309** ± *2.7*
	**24**	**29** ± *2.2*	**68** ± *8.4*	**200** ± *54.9*	**37** ± *6.5*	**68** ± *28.2*	**411** ± *270.8*	**33** ± *2.9*	**75** ± *19.9*	**72** ± *29.7*
IL1B	**1**	**139** ± *28.3*	**190** ± *0.7*	**3,157** ± *337.8*	**47** ± *4.3*	**53** ± *3.4*	**3,472** ± *1,110.2*	**4.6** ± *0.6*	**7.7** ± *1.5*	**3,176** ± *153.4*
	**3**	**348** ± *3.6*	**601** ± *12.4*	**3,830** ± *379.7*	**246** ± *7.9*	**388** ± *34.9*	**1,903** ± *316.6*	**84** ± *1.6*	**127** ± *23.9*	**1,070** ± *1.8*
	**24**	**204** ± *21.3*	**760** ± *143.5*	**1,525** ± *329.3*	**157** ± *13.3*	**412** ± *73.2*	**679** ± *277.6*	**119** ± *12.0*	**418** ± *55.6*	**97** ± *31.1*
IL6	**1**	**230** ± *72.1*	**440** ± *173.6*	**2,381** ± *269.1*	**152** ± *26.9*	**157** ± *57.7*	**801** ± *77.3*	**8.8** ± *1.9*	**14** ± *4.6*	**1,806** ± *283.5*
	**3**	**175** ± *31.1*	**138** ± *9.1*	**1,106** ± *175.9*	**299** ± *12.1*	**624** ± *162.6*	**1,631** ± *486.1*	**191** ± *39.4*	**221** ± *71.9*	**1,823** ± *187.7*
	**24**	**27** ± *7.1*	**46** ± *11.6*	**1,012** ± *388.4*	**32** ± *8.3*	**19** ± *12.8*	**1,389** ± *659.9*	**25** ± *3.1*	**40** ± *7.8*	**135** ± *43.0*
IL10	**1**	**12** ± *1.0*	**13** ± *2.2*	**8.4** ± *1.1*	**9.4** ± *1.9*	**9.0** ± *1.1*	**16** ± *5.6*	**2.3** ± *0.1*	**3.8** ± *0.7*	**11** ± *0.4*
	**3**	**17** ± *2.6*	**24** ± *1.3*	**78** ± *0.5*	**9.4** ± *1.2*	**13** ± *2.9*	**16** ± *1.0*	**5.3** ± *0.6*	**10** ± *2.7*	**19** ± *1.0*
	**24**	**2.6** ± *0.1*	**6.0** ± *1.2*	**18** ± *3.1*	**4.4** ± *0.4*	**4.4** ± *1.6*	**41** ± *26.8*	**2.3** ± *0.3*	**4.3** ± *0.2*	**10.0** ± *3.5*
CXCL2	**1**	**144** ± *26.1*	**167** ± *3.2*	**1,345** ± *199.2*	**95** ± *23.3*	**73** ± *0.5*	**2,087** ± *841.6*	**8.1** ± *0.7*	**8.2** ± *1.6*	**1,663** ± *0.6*
	**3**	**202** ± *12.7*	**244** ± *21.9*	**1,140** ± *148.4*	**183** ± *28.0*	**226** ± *45.4*	**586** ± *143.7*	**65** ± *2.0*	**78** ± *19.9*	**391** ± *16.6*
	**24**	**130** ± *14.1*	**246** ± *23.9*	**537** ± *140.9*	**186** ± *14.8*	**166** ± *49.4*	**575** ± *311.6*	**104** ± *19.0*	**175** ± *5.0*	**68** ± *20.4*
CXCL8	**1**	**21** ± *2.9*	**30** ± *2.3*	**258** ± *6.4*	**17** ± *4.6*	**19** ± *1.8*	**466** ± *126.6*	**5.1** ± *0.1*	**7.2** ± *2.0*	**366** ± *14.9*
	**3**	**36** ± *0.0*	**48** ± *0.2*	**187** ± *25.9*	**47** ± *13.0*	**60** ± *14.9*	**140** ± *33.2*	**22** ± *1.5*	**21** ± *4.8*	**124** ± *0.7*
	**24**	**20** ± *1.0*	**87** ± *22.4*	**54** ± *14.9*	**36** ± *0.3*	**59** ± *10.9*	**94** ± *47.4*	**22** ± *3.8*	**55** ± *2.3*	**29** ± *8.3*
CCL5	**1**	**64** ± *24.2*	**66** ± *5.8*	**1,130** ± *25.6*	**7.7** ± *1.6*	**8.5** ± *0.1*	**6,121** ± *1,833.4*	**13** ± *6.6*	**2.2** ± *0.2*	**1,227** ± *112.7*
	**3**	**216** ± *63.0*	**297** ± *9.2*	**5,941** ± *812.4*	**34** ± *8.4*	**55** ± *12.9*	**784** ± *133.5*	**34** ± *23.6*	**13** ± *3.7*	**255** ± *10.7*
	**24**	**79** ± *13.9*	**320** ± *9.9*	**1,871** ± *375.1*	**149** ± *6.3*	**265** ± *1.9*	**368** ± *215.5*	**66** ± *15.1*	**95** ± *12.1*	**27** ± *10.2*
CCL20	**1**	**192** ± *37.7*	**131** ± *5.4*	**1,029** ± *72.6*	**32** ± *8.9*	**25** ± *3.1*	**1,802** ± *672.6*	**3.7** ± *1.3*	**3.9** ± *1.1*	**1,526** ± *110.9*
	**3**	**619** ± *26.5*	**567** ± *17.9*	**6,062** ± *590.4*	**315** ± *97.2*	**336** ± *87.5*	**1,843** ± *335.4*	**102** ± *8.9*	**76** ± *21.4*	**927** ± *53.8*
	**24**	**46** ± *1.7*	**328** ± *10.6*	**1,734** ± *411.5*	**78** ± *7.2*	**184** ± *45.3*	**552** ± *397.5*	**47** ± *9.7*	**131** ± *29.2*	**38** ± *14.8*
NOS2A	**1**	**8.3** ± *0.6*	**11** ± *4.9*	**26** ± *2.3*	**5.7** ± *1.7*	**7.3** ± *0.1*	**273** ± *112.3*	**2.7** ± *0.1*	**2.5** ± *0.4*	**53** ± *0.4*
	**3**	**48** ± *0.7*	**111** ± *1.9*	**224** ± *39.4*	**54** ± *12.4*	**74** ± *10.2*	**108** ± *4.1*	**18** ± *4.4*	**18** ± *5.9*	**65** ± *0.6*
	**24**	**18** ± *0.6*	**67** ± *10.1*	**15** ± *4.3*	**48** ± *2.9*	**109** ± *20.8*	**25** ± *17.0*	**13** ± *1.2*	**26** ± *26.4*	**3.9** ± *3.9*
LAP	**1**									
	**3**		**not expressed**			**not expressed**			**not expressed**	
	**24**									
SIGIRR	**1**									
